# Vestibular anomalies and dysfunctions in children with inner ear malformations: A narrative review

**DOI:** 10.3389/fped.2023.1027045

**Published:** 2023-02-27

**Authors:** Davide Brotto, Marzia Ariano, Mosè Sozzi, Roberta Cenedese, Eva Muraro, Flavia Sorrentino, Patrizia Trevisi

**Affiliations:** ^1^ Section of Otorhinolaryngology – Head and Neck Surgery, Department of Neurosciences, University of Padova, Padova, Italy; ^2^ Department of Medicine, Camposampiero Hospital, Camposampiero, Italy

**Keywords:** inner ear malformations, hearing loss, vestibular function, balance, motor development

## Abstract

About 20% of children with congenital hearing loss present malformations of the inner ear. In the past few years much has been understood about the morphology and function of the anterior part of the labyrinth, since hearing loss may have a dramatic effect on the overall development of a child. Nowadays, for most of them, a chance for hearing rehabilitation is available, making hearing loss a treatable condition. The anomalies range from the lack of development of the whole inner ear to specific anomalies of isolated structures. Despite the frequent concomitant involvement of the posterior part of the labyrinth, this part of the inner ear is frequently neglected while discussing its morphology and dysfunction. Even though vestibular and balance function/dysfunction may have a significant impact on the global development of children, very little is known about these specific disorders in patients with inner ear malformations. The aim of this review is to summarize the available literature about vestibular anomalies and dysfunctions in children with inner ear malformations, discussing what is currently known about the topic.

## 1. Introduction

Inner ear malformations (IEMs) account for about 20% (1, 2) of the cases of congenital hearing loss. The spectrum of anomalies is wide, and despite the rarity of each malformation, they have been studied since the XVIII century (3) because of their impact on the hearing function. Since the first report by Mondini, a lot of anomalies have been described and classified until the latest and worldwide adopted classification proposed by Sennaroglu (2, 4). The development of new imaging techniques paved the way for different methods to describe these anomalies and to make them easier to understand (5, 6). In addition, the evolution of rehabilitation instruments for hard of hearing children enabled the research in this field, making the auditory dysfunction an addressable issue in most cases. Nowadays multiple strategies are available to restore hearing in these children, from cochlear implant to even brainstem implantation in selected patients (7).

In most cases, these malformations affect both the anterior part of the labyrinth and the posterior one, responsible for vestibular function (8). For example, patients with unilateral hearing loss seem to have a relevant prevalence of malformations of the inner ear also affecting the posterior part, in about 7% of cases (9). The anterior labyrinth has always caught more attention, because hearing loss affects the development of communication skills, especially in the first phases of life, causing severe disabilities. On the contrary, balance and motor development may be perceived as a less important problem, since visual and muscular feedback are believed to be able to minimize the impact on child evolution. Since today, good auditory performance is frequently achievable (by means of modern hearing aids, cochlear implants or brainstem implants) it is time to look beyond hearing, and focus our attention also on the impact of IEMs on vestibular function. Indeed, vestibular impairment might put the patients at higher risk of damage in specific professional or physical activities and can affect the overall quality of life.

The study of the vestibular function is reasonable since some authors suggest that some internal architecture (in terms of neural elements) might be preserved despite the presence of gross structural inner ear anomalies (10), thus implying that a residual function may be present. Conversely, even in cases in which the bony structure is preserved, anomalies of the membranous labyrinth can be present (such as in the cochleovestibular dysplasia): in these cases the macula of the saccule is degenerated, and the hair cells in the saccular macula are damaged, whereas the hair cells in the cristae of the three semicircular canals and utricular macula are preserved (11). So vestibular functioning should not be taken for granted even when the bony labyrinth seems to be morphologically normal.

Nonetheless, clinical practice shows that these patients frequently present delays also involving balance, neural and motor skills. Indeed, congenital vestibular dysfunctions may cause difficulty in staying seated, in getting up or walking (12). Specifically, problems like poor head control, the risk of head retroflexion and a delay in supporting the trunk by the legs due to a reduced labyrinthine muscle tone may be present in the first phases of life (13).

In addition, vestibular evaluation in children is less frequently performed due to poor compliance to the tests. Moreover, these posterior labyrinth anomalies can be part of syndromic frameworks like CHARGE (14) in which some authors suggest that they should be considered a characteristic feature (15).

Consequently, the aim of the present review is to summarize the available knowledge about the vestibular function/dysfunction in patients affected by each of the currently known IEMs. The different malformations will be presented according to the most frequently used and worldwide accepted classification proposed by Sennaroglu in 2002 (4) and revised in 2017 (see Table 1) (2).

**Table 1:** Sennaroglu's classification of inner Ear malformations.

Type of Malformation	Subgroups	Description
Complete labyrinthine aplasia (Michel deformity)	With hypoplastic or aplastic petrous bone	Absence of all the elements of the inner ear (cochlea, vestibule, semicircular canals, vestibular and cochlear aqueducts)
	With otic capsule	
	Without otic capsule	
Rudimentary otocyst	–	Small round cyst within an incomplete otic capsule without an internal auditory canal
Cochlear aplasia	With normal labyrinth	Absence of the cochlea, vestibule and semicircular canals might be present normal or altered
	With a dilated vestibule (CAVD)	
Common Cavity	–	Single, ovoid or round chamber, representing both cochlea and vestibule/semicircular canals without differentiation among them
Cochlear hypoplasia (external cochlear dimensions are smaller than normal)	Cochlear hypoplasia-I	Cochlea is like a small budd, vestibule and semicircular canals present with normal or abnormal shape
	Cochlear hypoplasia-II	Defective modiolus and interscalar septa, normal external shape and vestibule/semicircular canals present with normal or abnormal shape
	Cochlear hypoplasia-III	Short modiolus and less than 2 turns, reduced length of the interscalar septa and vestibule/semicircular canals present with normal or abnormal shape
	Cochlear hypoplasia-IV	Normal basal turn, middle and apical turns hypoplastic and located anteriorly and medially and vestibule/semicircular canals present with normal or abnormal shape
Incomplete partitions of the cochlea (differentiation of cochlea and vestibule, normal external dimensions)	Incomplete partition type I	The entire modiolus and interscalar septa are absent, resulting in a cystic cochlea. Vestibule and semicircular canals clearly distinguished from the cochlear part.
	Incomplete partition type II	Absence of the apical part of the modiolus and corresponding interscalar septa; middle and apical turns appear fused. Vestibule can also be enlarged. Semicircular canals often normal
	Incomplete partition type III	Absent modiolus but complete interscalar septa. The cochlea appears to be “empty”. Vestibule and semicircular canals are normal.
Vestibular malformations	–	Absent, hypoplastic or dilated vestibule. Cochlea is normal.
Semicircular canals malformations	–	Absent, hypoplastic or enlarged semicircular canals. Cochlea is normal.
Internal auditory canal malformations	–	Absent, hypoplastic or enlarged internal auditory canal. Cochlea, vestibule and semicircular canals are normal.
Enlarged vestibular aqueduct	–	Vertical and axial width larger than 1.5 mm at the midpoint between labyrinth and operculum

## 2. Methods

PubMed database was systematically screened up to July 2022 using the following free term search: vestibular AND (complete labyrinthine aplasia OR rudimentary otocyst OR common cavity OR incomplete partition OR cochlear hypoplasia OR enlarged vestibular aqueduct), obtaining 913 results. The literature search was performed by three authors independently (MA, DB and MS), without setting any temporal filter on publication dates. All the retrieved publications were evaluated to identify the most relevant ones, moreover the bibliography of the selected papers was scanned to find any useful article. Duplications or aggregations of pre-existing data were excluded; only articles in English and about humans were included.

**Figure 1:**
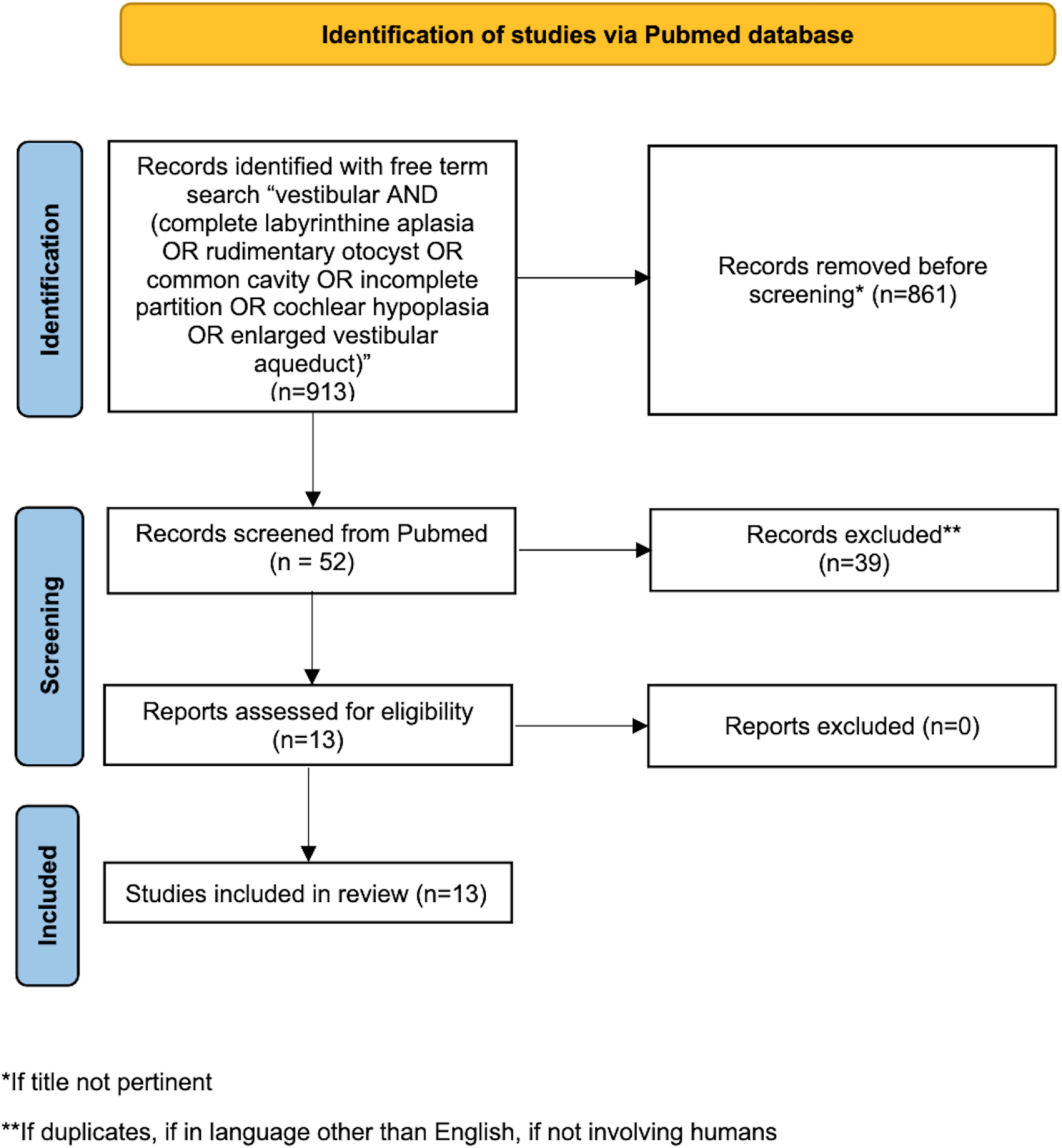
Flowchart for the identification of the studies in Pubmed database.

Every misalignment the authors had with regards to article eligibility was solved through discussion (see Figure 1 for the flowchart for identification of the studies *via* Pubmed database).

## 3. Discussion

The literature about the relationship between IEMs and the vestibular function is scattered and few studies are available.

As general considerations, a pair of old studies about CHARGE syndrome highlight that, in these patients, vestibular and semicircular canals anomalies are frequently detected and dysfunctions are extremely frequent (like always) in the limited cohorts investigated (17 and 5 patients respectively) (15, 16).

In particular Abadie et al. estimated that, while considering the timing to reach the same stages of the neuromotor development, CHARGE patients require 50% more time than peers without syndromic features, although this estimation is impaired by the presence of concomitant disabilities (visual and hearing impairment, central nervous system malformations, etc.) (15).

A delayed head control and delayed independent walking were observed in children with inner ear malformations from Inoue et al. too, however in this case the authors did not report specifically the type of malformations affecting the patients studied (17).

In these cases, absence or hypoplasia of semicircular canals was always associated with no measurable lateral canal function; in a case, normal morphology was associated with reduced canal function (15). Also, the older study by Admiraal et al. showed vestibular areflexia in the majority of cases (16).

More specifically, the available knowledge about vestibular function and IEMs will be summarized according to the latest classification system from Sennaroglu (2) that includes the following macro-categories: total labyrinthine aplasia, rudimentary otocyst, cochlear aplasia (with and without dilated vestibule), common cavity deformity, incomplete partitions (with three subtypes), cochlear hypoplasias (with 4 subtypes), isolated enlarged vestibular aqueduct, isolated vestibular abnormalities and anomalies on the internal auditory canal.

### 3.1. Total labyrinthine aplasia and rudimentary otocyst

**Figure 2:**
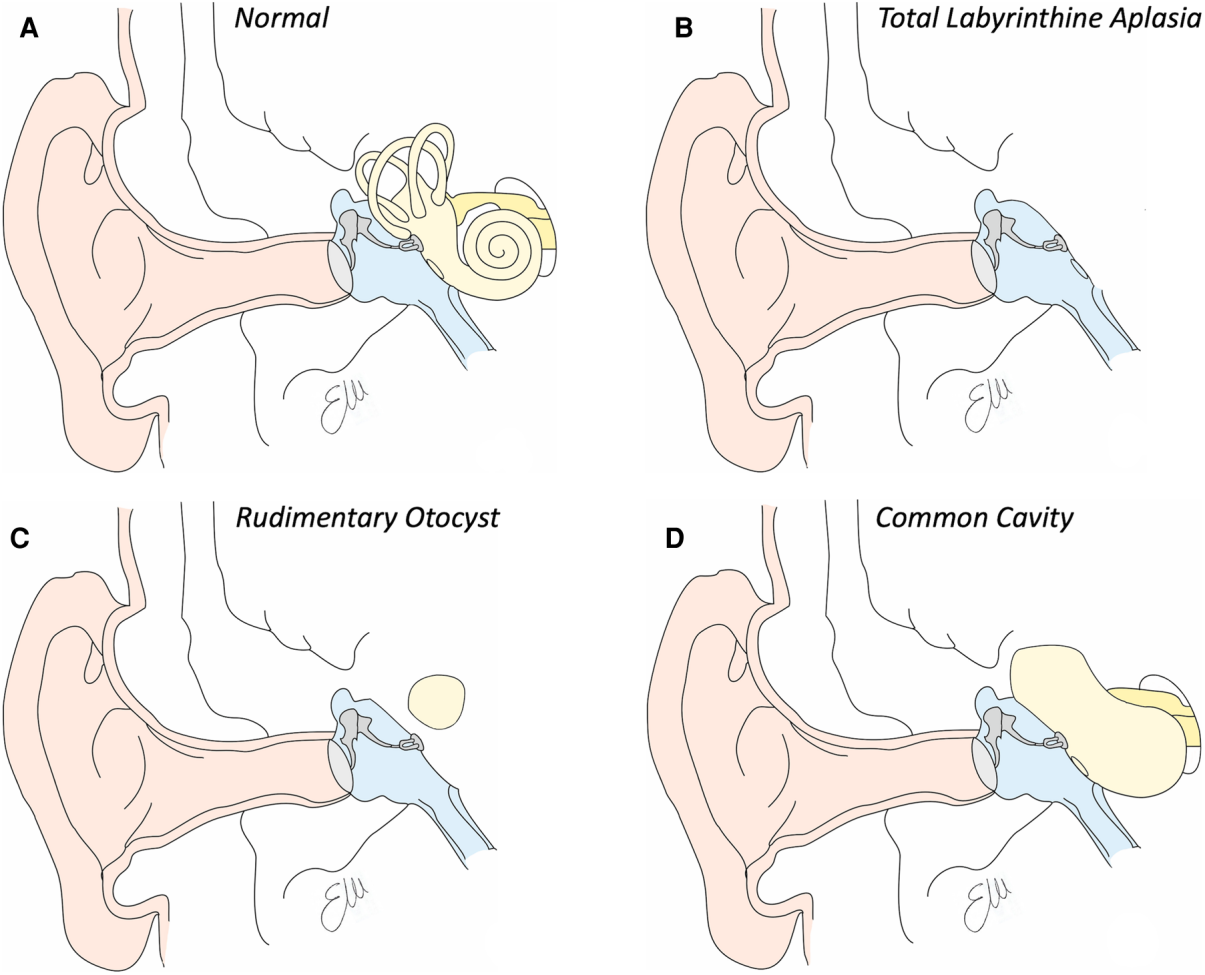
Drawings of inner ear structures in coronal view. (**A**) Normal anatomy. See in light yellow the bone labyrinth. (**B**) Drawing of total labyrinthine aplasia. Note the absence of the whole inner ear. (**C**) Drawing of a rudimentary otocyst, in which a single ovid cavity, representing the cochlear and vestibular part of the inner ear is within the otic capsule, without any communication with any internal auditory canal. (**D**) Drawing of a common cavity, a single cavity communicating with the internal auditory canal which presents a common cochleovestibular nerve.

Total labyrinthine aplasia is also known as Michel deformity, from the name of the surgeon Eugene Michel that described it. Noteworthy, the surgeon from Strasbourg was the one who described it in a book in 1863, but the first to ever report this anomaly was Jean Antoine Saissy in 1819 (3). This anomaly seems to account for around 1% of the IEMs and, when bilateral, it can be a sign of the LAMM syndrome (labyrinthine aplasia, microtia and microdontia) (18); also isolated cases are documented. The malformation is characterized by the absence of all the structures of the inner ear, both the anterior and posterior part; consequently, the middle ear (frequently normal) is divided from the encephalic spaces by a thin bone wall, as the otic capsule is basically missing as well as the internal auditory canal (see Figure 2B). Most probably the developmental arrest of the otocyst that determines the presence of this anomaly occurs at the third gestational week.

In these cases, the patient presents profound hearing loss with the only chance for auditory rehabilitation being the brainstem implantation.

Eugéne Michel also described for the first time the rudimentary otocyst, a malformation that is characterized by the presence of a single spherical cyst present into the otic capsule (see Figure 2C). This cavity is by definition not connected to the internal auditory canal, which may be absent or hypoplastic (it tends to contain only the VII and not the VIII cranial nerve. It is estimated that the prevalence of this malformation is also lower than 1% among all cochlear malformations. It is assumed that this picture develops as a consequence of the arrest of the embryological development of the otocyst around the end of the third gestational week, in which the otocyst fails to complete its migration towards the most medial part of the embryonic skull and therefore does not take contact with the forming internal auditory canal. This rounded structure represents both cochlear and vestibular structures. Just as the labyrinthine aplasia, hearing loss is profound and the only rehabilitative option is therefore brainstem implantation.

Regarding what is known about the vestibular function in these patients, unfortunately there is only one study in the literature, on a cohort of 10 patients with labyrinthine aplasia and/or rudimentary otocysts, using brainstem implant (19). In these patients, the oculo-vestibular reflex gain was significantly lower than in the subjects of the two control groups (with cochlear implant but without malformations and patients without hearing loss). In particular, the gain related to the posterior or lateral semicircular canal was nearly absent, while the gain related to the anterior ones on both sides was higher than that of the other canals. This result, according to the authors, could indicate a gain of the mechanical oculo-vestibular reflex in some patients with severe malformations of the inner ear. In the end, all patients presented with bilateral loss of vestibular function. None of the patients presented responses to ocular vestibular evoked myogenic potentials (oVEMPs) or cervical vestibular evoked myogenic potentials (cVEMPs) at 126 dB SPL on both sides, since in both labyrinthine aplasia and rudimentary otocyst otolithic organs are missing. Postural instability was also greater in patients with labyrinthine aplasia or rudimentary otocysts, compared to controls, but it was also detected (though to a lesser extent) even in children with cochlear implants (CIs) without malformations, compared to the group of healthy children (19).

### 3.2. Cochlear aplasia

**Figure 3:**
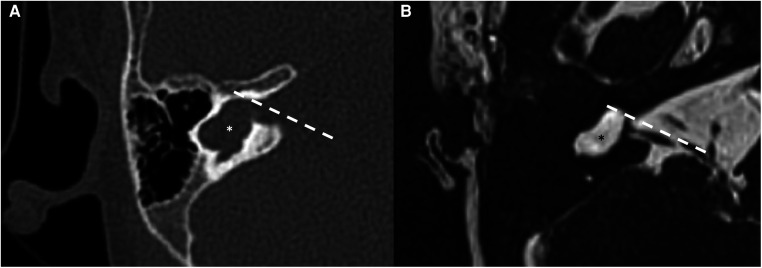
Bone-CT (**A**) and T2 weighted-MRI (**B**) imaging of a right cochlear aplasia. Note the complete development of the cystic cavity behind the axis of the internal auditory canal (dashed white line). The posterior labyrinth is cystic and it also represents the semicircular canals (asterisks).

Cochlear aplasia is a malformation characterized by the absence of the anterior portion of the labyrinth, the cochlear part (see Figure 3). The posterior labyrinth can be of normal morphology or with an enlarged vestibule determining the presence of two different entities: cochlear aplasia and cochlear aplasia with dilated vestibule. It is estimated that the prevalence of this malformation is less than 1% of all cochlear malformations. It is assumed that this malformation develops as a consequence of the arrest of the embryological development of the otocyst around the end of the third gestational week. In experimental mouse models, cochlear aplasia appears to be associated with mutations in the *Pax2* gene (20). Even in these patients, the hearing loss is profound and hearing rehabilitation is not possible with hearing aids. The possibility of performing cochlear implantation is controversial: according to some authors it can bring benefits, while according to others, the finding of cochlear aplasia is an indication for brainstem implantation (21). To date, there is no data in the literature regarding the vestibular function of patients with cochlear aplasia. As a pure speculation, it is reasonable to suppose that some kind of vestibular function should be present because only the anterior part of the labyrinth is believed to be missing, while the posterior should be at least in part preserved.

### 3.3. Common cavity

The common cavity is a cochlear malformation in which it is possible to identify a single cystic cavity, connected to the internal auditory canal, which represents both cochlear and labyrinth structures (see Figure 2D). The cavity has no internal subdivisions and may have a development that can be anteroposterior with respect to the axis of the internal auditory canal, only anterior (very rarely) or totally posterior (perhaps the most frequent form). The bottom of the internal auditory canal tends to be absent, making the structures of the inner ear subject to variations in intracranial pressure. It is estimated that the prevalence of this malformation is less than 1% of all cochlear malformations. It is assumed that this anomaly develops as a consequence of the arrest of the embryological development of the otocyst around the fourth gestational week. Even in these patients, the hearing loss is profound and hearing rehabilitation is not possible with hearing aids. In most cases, CI positioning is possible, with variable and still not completely predictable outcomes (21).

In the literature there are few studies evaluating vestibular function in patients with common cavity deformity. One is conducted in 8 patients, studied with a rotary chair. In this study the authors observed that in all patients the oculo-vestibular reflex was absent in the first year of life while it was clearly observable at least at 3 years. Head control and the ability to walk independently were still achieved, but later compared to controls without inner ear malformations. According to the authors, these results suggest that vestibular receptors are still present in the common cavity, precisely because the oculo-vestibular reflex develops around the age of 3 (13).

Another study investigates vestibular function before and after cochlear implantation using VEMPs. In two patients with common cavity VEMPs were present before and after cochlear implantation suggesting the presence of inferior vestibular neurons (22).

Some data are available about vestibular stimulation after cochlear implantation. Sennaroglu et al. reported a case of a patient with common cavity that showed nystagmus after four weeks from CI, suggesting a stimulation of the vestibular nerve fibers; the patient underwent cochlear implant fitting with the adjustment of T- and C-levels until no nystagmus was present and a good audiological outcome was reached (23).

### 3.4. Incomplete partitions

Incomplete partitions are malformations that are characterized by alterations in the internal morphology of the cochlea and account for about 40% of the malformations of the inner ear (24). The incomplete partitions are divided into 3 subgroups with different characteristics: incomplete partition type I, type II and type III.

#### 3.4.1. Incomplete partition type I

**Figure 4:**
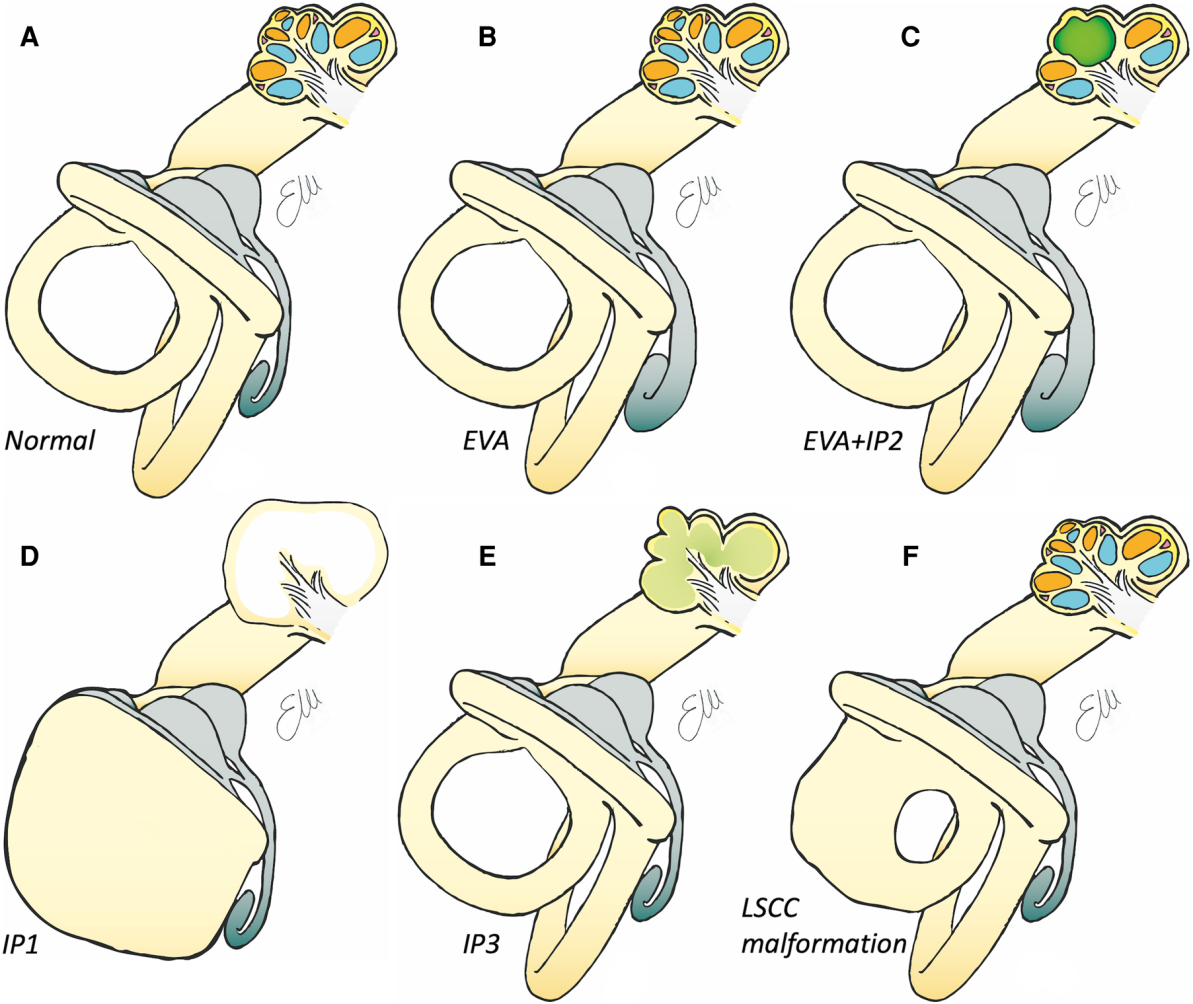
Drawings of inner ear structures showing both the auditory/anterior part (with an open cochlea) and the vestibular/posterior part. (**A**) Normal anatomy. See in gray the membranous labyrinth, with the endolymphatic sac with its sac-like shape, in light blue the scala tympani, in orange the scala vestibuli. (**B**) Drawing of enlarged vestibular aqueduct with the enlarged endolymphatic sac (in gray). (**C**) Drawing of a malformation in which there is the concomitant presence of the enlarged endolymphatic sac (in gray) and an incomplete partition type II, with the middle and apical turn (in green) of the cochlea are fused. (**D**) Drawing of an incomplete partition type I in which the cochlea is of normal dimensions but cystic, with the malformed posterior part of the labyrinth. (**E**) Drawing of an incomplete partition type III, in which the cochlea appears to be without internal architecture with normal (but overall elongated) external shape. (**F**) Drawing of an isolated malformed lateral semicircular canal.

**Figure 5:**
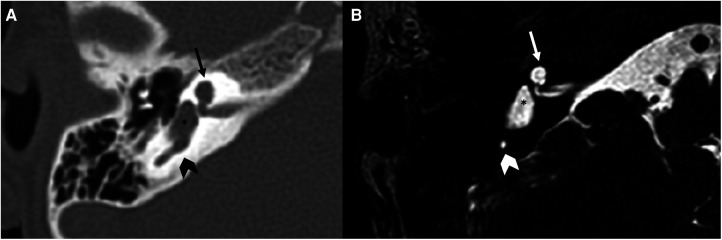
Bone-CT (**A**) and T2 weighted-MRI (**B**) imaging of a case of right incomplete partition type I. The anterior and posterior parts of the labyrinth are clearly divided. The anterior part is cystic (arrows), the posterior is dysplastic, although the vestibule (asterisks) is recognizable as well as a part of the posterior semicircular canal (arrowheads).

Among the incomplete partitions, certainly the one with the worst morphological and functional features is the incomplete partition type I (see Figures 4D, 5). In this type of malformation, the cochlear portion of the inner ear has a cystic morphology, clearly separated from the posterior portion which may be present with a normal morphology or altered in a series of ways, variably affecting the semicircular canals. The prevalence of this malformation is estimated to be around 2% of all cochlear malformations (2). It is assumed that this anomaly develops as a result of the arrest of the embryological development of the otocyst around the fifth gestational week. Even in these patients, hearing loss is profound and hearing rehabilitation is not possible with hearing aids. In most cases, the execution of the CI is possible, with good benefit if the cochlear nerve can be identified on MRI (2).

#### 3.4.2. Incomplete partition type II

**Figure 6:**
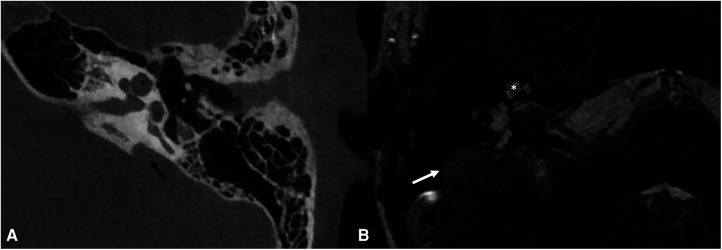
Bone-CT (**A**) imaging of a case of left incomplete partition type II and enlarged vestibular aqueduct (black arrow). T2 weighted-MRI (**B**) imaging of a case of right incomplete partition type II with enlarged endolymphatic sac (white arrow). Note in both images the presence of the upper part of the cochlea that appears to be globose (asterisks). The incomplete partition type II and the enlarged vestibular aqueduct are frequently both present in the same patient.

The incomplete partition type II was first described in 1791 by Carlo Mondini and is characterized by a normal morphology of the basal gyrus of the cochlea, and a middle and apical gyrus that appear fused and globose (see Figures 4C, 6) (3). This type of malformation is frequently associated with another malformation of the labyrinth: the enlarged vestibular aqueduct. In some cases, there are malformations of the vestibule and semicircular canals. In most cases the malformation is bilateral (25).

It is assumed that this anomaly develops as a consequence of the arrest of the embryological development of the otocyst around the VII gestational week. In these patients, the hearing loss is of a mixed type, initially of a mild-moderate degree that may remain stable over time or progressively worse, step by step (26). Vestibular symptoms can be associated with hearing impairments. In a recent study, more than 70% of patients demonstrated vestibular symptoms, of which 17% had bilateral vestibular areflexia on caloric stimulation while 10% showed no signs of vestibular dysfunction on caloric stimulation (25). In Pendred's syndrome, associated with mutations of the *SLC26A4* gene, the incomplete partition type II constitutes a characterizing element of the malformative spectrum, together with the presence of an enlarged vestibular aqueduct and thyroid goiter (27).

#### 3.4.3. Incomplete partition type III

**Figure 7:**
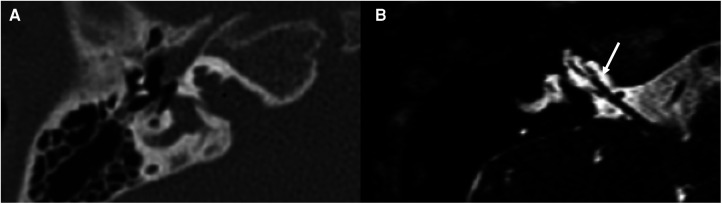
Bone-CT (**A**) and T2 weighted-MRI (**B**) imaging of a case of right incomplete partition type III. The cochlea appears to be without internal architecture, with the cochlear nerve clearly visible within (white arrow). The posterior labyrinth is almost always normal, but the absent fundus of the internal auditory canal (asterisk is the position in which it should be present in normal cochleae) can cause a third-window effect causing potential vestibular symptoms.

Incomplete partition type III is the only malformation of the inner ear for which X-linked inheritance linked to the mutation of the *POU3F4* gene is recognized (28). It is characterized by a normal external morphology of the cochlea with the absence of internal architecture, in particular the modiolus and the bottom of the internal auditory canal are absent. The vestibule and the semicircular canals have normal morphology (see Figures 4E, 7). This type of malformation represents less than 1% of inner ear malformations and can be associated with hypothalamic dysmorphism (29).

There is very little information in the literature regarding vestibular function in patients with incomplete partitions. The only available study in the literature reports that all patients with incomplete bilateral partition have a delay in the development of postural control (measured using the Bruininks-Oseretsky Test of Motor Proficiency Second Edition). Among the various partitions, however, patients with incomplete partition type II have better values than those with partition type I or type III. According to the authors, given the potential consequences, patients with bilateral incomplete partition could benefit from early vestibular rehabilitation support to accelerate the development of an adequate postural control (24). Another study, investigating the presence of VEMPs in patients with IEMs, showed no VEMPs before the CI surgery in all patients with incomplete partition type I and II, but the VEMPs were present after CI activation with CI on (22). The authors, specifically regarding the common cavities studied in the paper, suggest that in these cases the sensory cells of both saccule and inferior vestibular neurons may be present (22).

### 3.5. Cochlear hypoplasia

Cochlear hypoplasia is a large group of alterations in which the size of the cochlea is reduced compared to normal subjects by 2 standard deviations (mean values for hypoplasia: mean height of the cochlea in coronal vision 3.45 mm, height of the apical gyrus 3.07 mm and width 2.14 mm in axial images, length of the basal gyrus 8.13 mm) (30). This category of malformations is divided into 4 subgroups. Type I hypoplasia is identifiable as small cystic cochleae with no internal architecture (similar to type I incomplete partition in terms of shape, but reduced in dimensions). It is assumed that the arrest of embryological development in these patients occurs around the VI-VIII gestational week. Type II hypoplasia has cochlear dimensions below the norm, with incomplete modiolus and interscalar septa, but a normal external profile. Alterations of the vestibular aqueduct and the vestibule may be present simultaneously. Also, in this case it is assumed that the arrest of embryological development occurs around the VI-VIII gestational week. In type III hypoplasia, the cochlea is smaller than normal with a reduced number of turns (less than 2 turns and short modiolus). Usually the vestibule and semicircular canals are hypoplastic. Also, in this case it is assumed that the arrest of embryological development occurs around the VI-VIII gestational week. In these three types of malformation, the degree of hearing loss is profound, and cochlear implant is an available option, with the appropriate surgical precautions in consideration of the specific morphology.

Type IV hypoplasia, on the other hand, is characterized by a cochlea of normal size in the basal turn, but with the middle and apical turns extremely hypoplastic and located anteriorly, rather than in the usual position. In these cases, patients have moderate to severe-profound hearing loss. It is assumed that this type of malformation is caused by a developmental arrest between the X and XX gestational weeks (2). No information is available about the posterior part of the labyrinth associated with this type of malformation.

Vestibular function in patients with cochlear hypoplasia was investigated by Kimura et al. that studied the relationship between vestibular function and gross motor development in children with inner ear malformations. The results of the study show that children with inner ear malformations, including cochlear hypoplasia, presented a significantly reduced response in the rotational chair test, and head control and independent walking were significantly delayed compared to a control group (31).

### 3.6. Enlarged vestibular aqueduct

**Figure 8:**
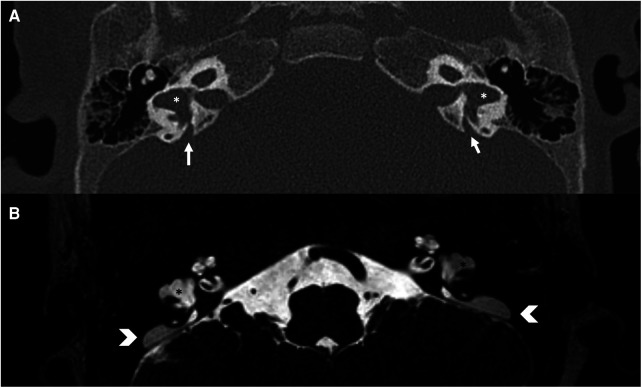
Bone-CT (**A**) and T2 weighted-MRI (**B**) imaging of a case of bilateral enlarged vestibular aqueduct (white arrows) and bilateral dysplastic lateral semicircular canals (asterisks). In CT the vestibular aqueduct is enlarged (white arrows), in MRI it is possible to observe the large endolymphatic sac within (white arrowheads) and in the middle cranial fossa.

Enlarged vestibular aqueduct (EVA) is one of the most commonly identified malformations of the temporal bone (25). It is reported to be the most common IEM associated with hearing loss in children, often occurring bilaterally (25), and probably the one about which the greatest amount of information is available (see Figures 4B, 8).

The exact prevalence in the adult and pediatric population is not yet precisely defined in literature. According to Song et al., EVA can be found in up to 13% of overall patients with sensorineural hearing loss (SNHL) and in up to 32% of pediatric patients with non-syndromic SNHL (32). According to other authors, vestibular aqueduct anomalies are identified only in 14% of pediatric SNHL patients, although this data is probably underestimated (33).

Radiologically, according to Valvassori and Clemis' definition, dating back to 1978, EVA is defined as a vestibular aqueduct diameter greater than or equal to 1.5 mm at the midpoint or greater than or equal to 2 mm at the operculum (34), while other authors proposed more recent but less used criteria (width > 1 mm at the midpoint and/or an opening width > 1.9 mm at the operculum) (32). It can be diagnosed with computed tomography (CT) scans, showing the enlargement of the vestibular aqueduct, as well as and T2-weighted magnetic resonance imaging (MRI), which on the contrary provides information about the membranous structures namely the endolymphatic duct and the endolymphatic sac (35).

During embryological development each part of the inner ear follows distinct growth trajectories. For example, the development of the labyrinth derives from the otocyst during the 4th week of gestation and most of the labyrinth reaches adult size by the 25th. On the other hand, the vestibular aqueduct grows throughout all the embryonic life reaching maturity later, according to some authors some months after birth (36).

Consequently, the enlargement of the vestibular aqueduct can be observed isolated or in association with cochlear and modiolar defects (in this case the diameter is reported to be larger than in isolated EVA) (36). This is known since the XVIII century: indeed, what is called the “Mondini malformation” is the combination of EVA, enlarged vestibule and cochlear incomplete partition type II (25). Nowadays it is demonstrated, even radiologically, that vestibular malformations, such as EVA, are often associated with other inner ear anomalies in up to 60 to 88% of patients (33). Cochlear dysplasia associated with EVA can be a very common anomaly with an incidence up to 57.5%. Modiolar hypoplasia associated with EVA has been reported to occur between 8.1% and 71% while modiolar hypoplasia can be associated with EVA in about 49% of patients (37).

Human inner-ear malformation types have been characterized by Dhanasingh et al. (6) using 3D segmented models: by doing a volumetric analysis in inner ears affected by EVA, they found out a difference in volume comparing a “normal” cochlea and the cochlea with EVA, 87.55 mm^3^ and 103.73 mm^3^ respectively. They also observed that the internal auditory canal (IAC) of normal anatomy cochlea appears to be wider than IAC from enlarged vestibular aqueduct syndrome (EVAS) (38). This anomaly might be a sporadic observation based on the limited number of cases examined in the cohort, or a characteristic feature (maybe due to the different pattern of diffusion of the intracranial pressure imposed by the enlarged aqueduct during embryonic life) that needs to be confirmed in future studies.

It is still unclear whether vestibular malformations correlate or cause cochlear abnormalities and if this could be associated with the entity of clinical symptoms. Sennaroglu et al. hypothesize that an increased fluid pressure through the EVA during ontogenesis could influence cochlear morphology, determining a dilatation of the vestibule and the cystic evolution of the apical part of the cochlea, leading to the so-called Mondini malformation (25, 39). On the other hand, some authors dispute, with some reasons, that pressure does not have a specific role in causing the presence of incomplete partition type II in cases of EVA (40).

Regarding genetic features which can be associated with anatomical abnormalities, it is speculated that a more severe impairment of pendrin (namely if a biallelic SLC26A4 mutation is present) may correlate with a higher inner ear fluid pressure, worse hearing thresholds, and more severe malformations such as wider diameters in EVA and Mondini Malformation, as confirmed by Forli et al. (25). In their study, patients with biallelic SLC26A4 mutations were more likely to present Mondini Malformation than isolated EVA. EVA can also be associated with an enlarged endolymphatic duct and sac, specifically evaluated using MRI imaging, being described so in 32% of children with non-syndromic SNHL and in 55%–94% of overall patients with EVA (41).

EVA can be nonsyndromic (NSEVA or *DFNB4*) or it can be associated with syndromic conditions, such as Pendred Syndrome, distal renal tubular acidosis, branchio-oto-renal syndrome (BOR) or Waardenburg syndrome.

From the available literature, it appears that vestibular symptoms are not significantly associated with the size of the vestibular aqueduct (32).

Conversely, there appears to be a linear relationship between EVA and progressive SNHL: patients with bilateral EVA and SLC26A4 mutations usually have a higher rate of SNHL. Moreover, unilateral EVA is not a strictly unilateral process because hearing loss and vestibular hypofunction have been observed also on the contralateral side, not affected by EVA (42).

Up until now many hypotheses have been made about the mechanism causing hearing and vestibular disorders in EVA patients, mainly regarding the impact that inner ear fluids pressure and the resulting hydroelectrolytic imbalance determine on inner ear membranous structures. According to Kaya et al. the loss of both vestibular type I and type II hair cells in Mondini dysplasia (in post-mortem specimens), was statistically significant in all the semicircular canals (43). Indeed, it is postulated that the pressure of the cerebro-spinal fluid (CSF) in the intralabyrintic spaces (*via* the the EVA) might cause direct damage to inner ear structures or to the inner ear blood supply, maybe causing cellular loss (44).

Keeping that in mind, there are many trigger mechanisms that alter inner ear fluid pressure and that in these patients can be associated with the beginning or with the progression of cochlear and vestibular symptoms, such as head trauma, Valsalva maneuver, physical exercise, barotrauma or high fever (25). An higher compliance of the enlarged vestibular aqueduct (just like a higher compliance in Reissner's membrane results in endolymphatic hydrops) (44), causes the vestibular duct to lose its “anti-reflux valve” role in its rugose midpoint as demonstrated by Han et al. (45). In particular vestibular dysfunction may be precipitated by the reflux of hyperosmotic fluid into the basal end of the cochlea resulting in an osmotic and chemical imbalance (probably also explaining the worsening course of high frequencies hearing threshold over time in EVA patients).

In addition the high inner ear fluid pressure can limit the stapes movement causing the conductive component of the hearing loss often observed in EVA patients (25).

Another mechanism that may explain the conductive component of the hearing loss is the third-window effect induced by EVA (see later for an explanation) (25).

Clinically speaking EVA has been associated with various patterns of SNHL: from mild to profound degree, with different age of presentation and with abrupt onset, fluctuating or progressive. While many studies discuss extensively about this topic little is still known about the vestibular symptoms associated with this kind of malformation.

As reported in literature the overall incidence of vestibular symptoms in EVA subjects vary from 4% (33) to 100% (46, 47), and they range from severe episodic vertigo to occasional unsteadiness in adults, whereas incoordination and imbalance predominate in children, compared with controls (38, 47).

This could be explained by some sort of bias from the patients in reporting vestibular symptoms associated with their condition, as they seem less relevant compared to the auditory ones. The difficulty is even greater in children (EVA subjects are usually diagnosed at an early age) due to poor children accuracy and poor compliance in reporting balance disorders and subject themselves to diagnostic testing: a deficiency in vestibular end organs may be indirectly suspected in the pediatric if delayed motor skills (e.g., poor head and neck control, delays in independently sitting or walking), clumsy behaviors, and frequent falls are noted (43).

Also, about the type of vestibular symptoms reported, there is no extensive data in the literature. Song et al. (32, 48) propose that all EVA patients with vestibular symptoms should undergo evaluations for the development of secondary benign paroxysmal positional vertigo (BPPV) in regards with the high incidence of this pathology (18.2%) in their cohort, probably from dislodgement of the otoconia from the utricle caused by inner ear pressure imbalance. According to other authors, BPPV in EVA patients could be spontaneous, but a distinguishing feature is the frequent sensitivity to mechanical triggers such as minor head trauma or vigorous self-rotation, so patients should be advised to avoid these kinds of situations (46).

Another topic of interest could be the relationship between cochlear implantation and vestibular symptoms in EVA patients. Although Reynard et al. (41) suggest that children with EVA are at greater risk of vestibular loss during cochlear implantation compared to controls, a “spontaneous” vestibular compensation is still possible, as shown by other studies (49). Nonetheless, the same authors strongly recommend that even pediatric EVA patients should be advised to undergo intensive specialized physical therapy after cochlear implantation, and that, if CI is needed bilaterally, this surgical procedure should be performed sequentially, to avoid severe vestibular ataxia in case of post-surgical bi-vestibular failure (41). Noteworthy, what is recommended by the above-mentioned authors are based on the limited studies available, and studies on larger cohorts are necessary to adopt these suggestions as general recommendations.

Regarding diagnostic findings in EVA patients, curiously there is a discrepancy between caloric testing and video head impulse test (vHIT) as the former usually shows unilateral caloric weakness while the latter is normal (50).

Jung et al. (50) hypothesize that the low rate of abnormal findings in the vHIT could imply that vestibular dysfunction in EVA might either be affected only by low-frequency stimuli (explored by caloric testing), or that central compensation for VOR impairment could differ according to the frequency of the stimulus.

Abnormal caloric findings, however, should not be taken for granted in EVA patients and in Mondini's deformity because in these conditions, the horizontal SCC (the one responsible for the evoked vestibular response after bithermal water- or air-caloric irrigation) and ampulla have both a pouch-shaped appearance in which the cupula is not enclosed by a membranous wall, and the fluid movement caused by caloric stimulation may not create a pressure gradient sufficient to deflect the cupula (48). Moreover, this leaves the membranous labyrinth vulnerable to sudden fluctuations in CSF pressure, as previously reported (51).

Also, it should be pointed out a discrepancy between reported vestibular symptoms and the results of vestibular testing: these tests are often abnormal even if no clinical vestibular complaint has been made by the patients, highlighting the importance of performing vestibular function testing regardless of whether there are reported symptoms (34, 51).

Another diagnostic testing which can be useful in EVA patients is the cVEMP test, although few studies are available. cVEMP test uses electromyography in the neck muscles, measuring a vestibulospinal reflex mediated through the saccule and the inferior vestibular nerve, in response to a loud auditory stimulus.

The characteristics of cVEMP in EVA include lower thresholds and higher amplitudes: the abnormally low cVEMP threshold suggests a “third-window” effect in this pathologic condition.

In fact, EVA could act as a “third” mobile window, in which air-conducted sounds are shunted away from the cochlea to the vestibule, just like superior semicircular dehiscence syndrome in which cVEMPs findings are very much alike to EVA. This mechanism, could make the peripheral vestibular system, such as the semicircular canals and otolith organs, more excitable and sensitive than normal, as explained by Zhou et al. (48, 52, 53). In the largest study on EVA patients all the subjects with absent cVEMPs presented vestibular complaints (53).

Up until now few studies focus their attention on oVEMPs, instead. For example, in a case report of a patient with EVA, Taylor et al. (54) note that despite cVEMP amplitudes and thresholds were normal for both ears across all frequencies of stimulation, augmented oVEMP amplitudes were registered, maybe making oVEMPs a more sensitive indicator of vestibular hypersensitivity than cVEMPs.

To sum up, vestibular symptoms in EVA patients vary both in prevalence and typology across the different studies included in this review. These studies are often contradictory especially regarding vestibular testing results and prevalence of symptoms reported, and many of them do not have extensive numbers of patients.

### 3.7. Isolated malformations of the semicircular canals

In the literature, isolated malformations of the semicircular canals and vestibule are also described, which may appear hypoplastic or dysplastic (see Figures 4F, 8). They are often coincidental findings.

There is few literature on the subject, given the rarity of these dysmorphisms, and none of it regarding the pediatric population.

From an audiological point of view, patients with lateral semicircular canal (LSCC) dysplasia present in around 60% of cases a certain amount of hearing loss, specifically 39% present SNHL and 12% mixed hearing loss. The prevalence of hearing loss rises up to 80% in patients with bilateral malformation (54).

Vestibular function was investigated in 15 patients with LSCC dysplasia/aplasia; 6 of them had bilateral LSCC dysplasia/aplasia combined with other inner ear anomalies. On a caloric test, patients with isolated LSCC dysplasia showed a 51.8% ± 29.3% level of canal paresis, whereas patients with bilateral LSCC dysplasia/aplasia presented bilateral vestibular loss (55).

Vestibular function was also studied in children with semicircular canal aplasia using rotational chair test and all subjects had no response, the same author studied on the same sample the gross motor development and found that acquisition of head control and independent walking was significantly delayed (56).

### 3.8. Isolated anomalies of the internal auditory canal

Alterations to the internal auditory canal are extremely rare entities that can be associated with alterations relating to the presence, course and morphology of the eighth cranial nerve. However, their clinical significance, from a vestibular and in some ways also audiological point of view, remains to be defined and no literature is available about this topic.

## 4. Conclusion

Only few studies address the topic of vestibular function in patients with IEMs. Consequently, the knowledge that can be extrapolated is scattered and at this stage controversial. The available studies present severe limitations, such as the restricted number of patients, the heterogeneity of the cohorts and the methods used.

Malformations affecting the posterior part of the labyrinth are a relevant feature when considering patients with hearing loss, they can be unilateral or bilateral, the spectrum of these anomalies is wide and the impact on the clinical course is not fully characterized yet. Even when considering EVA patients (about which the greatest amount of literature is available), some pieces of the puzzle are still missing, despite being the better described group.

Many issues are still unsolved, such as the relationship between the morphology of the anomalies and the consequences in terms of vestibular function, the impact on the overall balance and motor development and the relationship between cochlear implant function and vestibular responses.

Only future studies on larger and homogeneous cohorts with standardized methodology will unveil the secrets of the impact of these anomalies on vestibular function.
